# A critical look at the prediction of the temperature field around a laser-induced melt pool on metallic substrates

**DOI:** 10.1038/s41598-021-91039-z

**Published:** 2021-06-09

**Authors:** Yi Shu, Daniel Galles, Ottman A. Tertuliano, Brandon A. McWilliams, Nancy Yang, Wei Cai, Adrian J. Lew

**Affiliations:** 1 Department of Mechanical Engineering, Stanford University, Stanford, CA 94305 USA; 2 Oak Ridge Associated Universities, Oak Ridge, TN 37830 USA; 3 DEVCOM Army Research Laboratory, Aberdeen Proving Ground , MD 21005 USA; 4 Sandia National Laboratories, Livermore, CA 94550 USA

**Keywords:** Mechanical engineering, Computational science

## Abstract

The study of microstructure evolution in additive manufacturing of metals would be aided by knowing the thermal history. Since temperature measurements beneath the surface are difficult, estimates are obtained from computational thermo-mechanical models calibrated against traces left in the sample revealed after etching, such as the trace of the melt pool boundary. Here we examine the question of how reliable thermal histories computed from a model that reproduces the melt pool trace are. To this end, we perform experiments in which one of two different laser beams moves with constant velocity and power over a substrate of 17-4PH SS or Ti-6Al-4V, with low enough power to avoid generating a keyhole. We find that thermal histories appear to be reliably computed provided that (a) the power density distribution of the laser beam over the substrate is well characterized, and (b) convective heat transport effects are accounted for. Poor control of the laser beam leads to potentially multiple three-dimensional melt pool shapes compatible with the melt pool trace, and therefore to multiple potential thermal histories. Ignoring convective effects leads to results that are inconsistent with experiments, even for the mild melt pools here.

Metallurgical characteristics, mechanical properties, and corrosion resistance of additively manufactured metallic parts are known to be strongly affected by thermal histories^1^, such as the time evolution of temperature, temperature gradient, cooling rate and solidification front speed. In Selective Laser Melting (SLM), such thermal histories are induced by a laser beam moving on the top surface of a substrate submerged in a powder bed. However, the thermal history underneath a substrate’s surface is very difficult to measure^2^. Computational models can help gain information about it^2^.

The complex multi-physics of the problem raises the question of how well thermal histories in the substrate can be extracted from a computational model. In the solid regions of the substrate, the temperature history is defined by heat conduction, the way heat can escape the substrate through its boundaries, and most importantly, the time-dependent shape of the melt pool boundary. This boundary is diffusely located somewhere between the liquidus and solidus isotherms, and their evolution defines the solidification front speed. In contrast, in the liquid or melted region of the substrate, heat transport takes place through both heat conduction and convection, i.e., heat transported by the moving fluid particles. The motion of the fluid is largely driven by surface tension gradients induced by temperature gradients (the Marangoni effect), and the recoil-pressure from evaporating fluid particles on the free surface of the fluid. The fraction of the energy of the laser beam absorbed by the substrate is a result of both the temperature-dependent absorptivity of the metal and the geometry of the liquid surface, typically ranging from 20–30% for mild melt pool surfaces to above 80% in the presence of a cavity generated by a keyhole^3^. In the latter case, the motion of the liquid surface is often fast and non-smooth, so mixing and heat transport are enhanced. This behavior introduces a large shadow of uncertainty on how well the flow and heat transport in the melt pool can be computed under such conditions.

A key observation about the problem of computing the thermal histories in the solid region of the substrate gives rise to the question we examine in this paper. The observation is that in a frame in which the temperature distribution is steady, the heat conduction problem in the substrate is well-defined if the three-dimensional (3D) shape of the melt pool boundary is known, as we discuss later. Therefore, if a model is calibrated to reproduce the 3D shape of the melt pool from experiments, then the thermal history in the solid region would depend largely on the details of the heat conduction in the solid, such as latent heat for phase changes, and temperature-dependent specific heat and thermal conductivity, and less on the more uncertain features of the fluid motion. However, the 3D shape of a melt pool has not yet been measured; some two-dimensional (2D) projection of its shape is obtained instead. For example, through X-ray radiography^4,5^, or more commonly, by examining 2D traces of melt pool boundaries revealed after etching, c.f. Fig. 1.

Traces of laser-induced melt pools without a keyhole usually take shapes that resemble parts of ellipses, and a roster of models with different degrees of complexity can produce traces of this form^6^. This includes several models in which heat transport takes place only through heat conduction and are based on analytical expressions, beginning from those^7–9^ rooted in the solution to a moving pointwise heat source by Rosenthal^10^, to others than account for distributed heat sources^11–14^, temperature-dependent properties^15^, phase changes^16,17^, or finite speed thermal transport^18^. It also includes models with both conductive and convective heat transport driven by the Marangoni effect but without deformation of the melt pool top surface^19–22^, as well as models that allow for vertical motion of the melt pool and account for the recoil pressure to capture the formation of keyholes^23–25^. These models are often compared against single line weld experiments^20,22,26–29^, in which a heat source (e.g., a laser beam) is moved at a constant speed and power over the flat surface of a metallic substrate, see Fig. 1. Parameters that need to be adjusted or measured to match the dimensions of the 2D melt pool trace include the power source (laser beam/arc radius^23,26,30–34^), the fraction of energy absorbed by the substrate, typically extracted from the literature, and effective material properties (viscosity^27,29,35^ and thermal conductivity^27,29,33,35^) of the molten material to ignore convective effects and account for the observed enhanced thermal transport, sometimes attributed to turbulence. Apart from some occasions in which the heat source was characterized and included in the computational model, e.g.^36^, it is customary to assume the form of the power density^23,26,30–34^. The end result is that 2D melt pool traces can be acceptably reproduced by these models, at least on a per-experiment basis. It is known that multiple combinations of the laser’s speed and power can give rise to the same 2D melt pool trace^29^.

Given the availability of 2D melt pool traces to calibrate models to estimate the thermal history in the solid substrate, the question we seek to analyze here is: Is it possible to recover the 3D shape of a melt pool with a computational model that reproduces the 2D melt pool trace? The answer to this question hinges on whether there exist significantly different 3D melt pool shapes compatible with experimental conditions that produce similar 2D melt pool traces. This question is relevant because, if the 3D shape of a melt pool can be uniquely recovered (or a good approximation thereof), then based on the observation above, it would be possible to estimate the thermal history in the solid substrate from the model. In contrast, the existence of multiple 3D melt pools compatible with experimental conditions would give rise to incertitude in the computed thermal histories: a different one per 3D melt pool shape.

Our results here suggest that this is possible under certain conditions. First, we need good enough characterization and control of the power density distribution of the laser beam over the substrate. Second, convective heat transport effects cannot be ignored and should be accounted for. In particular, and for most laser beams, this implies that fine control of the position of the substrate surface along the optical axis is needed. Additionally, this means that, in modelling, (a) substituting the experimentally-measured power density distribution and instead assuming its form, such as Gaussian, is not generally justified, and (b) even in melt pools traditionally deemed to be dominated by conductive heat transport, convective effects play an important role and should be included. This is in some way unfortunate, since models that ignore convective effects are substantially less onerous in terms of time and computing resources to compute thermal histories in the solid region^6^.

**Figure 1:**
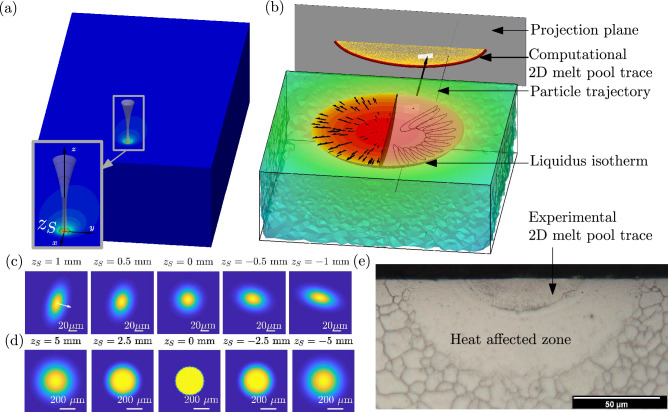
(**a**) We performed single-line weld experiments, in which the laser beam melts a metallic substrate along a straight line. Shown is the simulation domain we adopted, which is a box that moves together with the laser. An enlarged view of the temperature field is shown in the inset, together with a sketch of the power density distribution that would be shone on the sample surface at different locations $z_S$ along the optical axis of the laser. (**b**) Once the temperature field is obtained, the liquidus isotherm is computed and projected onto a surface perpendicular to the laser velocity. The boundary of the projected region (below the top surface) is the computational 2D melt pool trace. (**c**,**d**) Power density distributions of the astigmatic Gaussian (G) beam (in (**c**)) and the multi-Gaussian (MG) beam (in (**d**)), as a function of sample surface location $z_S$ along the optical axis used in our models. The sample surface is at the beam waist when $z_S=0$. The characterization experiment results can be found in the Supplementary Information. (**e**) An optical image of the etched section from the experiment ${\hbox {SG}}_3$ showing the experimental 2D melt pool trace, identified as the curve that separates the two regions with different apparent feature sizes and morphology.

To examine this question, we performed three sets of single-line weld experiments (see Fig. 1) on two different substrates, 17-4PH stainless steel (SS) and Ti-6Al-4V, with two different laser beams with different spot sizes and power density distributions. Specifically, an astigmatic Gaussian beam (G) with spot size varying around $60~\upmu \text {m}$, and a multi-Gaussian beam (MG) with spot size varying from $330~\upmu \text {m}$ to $625~\upmu \text {m}$ with an approximately flat top distribution at the focal plane^37^. We measured the power density distribution of the two laser beams as a function of the distance along the optical axis (Fig. 1 and Supplementary Information). We designed the experimental conditions (laser power and scanning speed) with the goal of obtaining melt pools in which the into-the-substrate motion of the melt pool surface is rather small, so that it does not define the melt pool shape, see Table 1. In particular, we purposefully avoided the appearance of keyholes. Substrates were cut along planes orthogonal to the laser trajectory, and the exposed sections were etched to reveal the region of the substrate that melted and re-solidified (Fig. 1).

Additionally, we considered two computational models: (a) a model in which heat transfer happens through heat conduction only (Fourier law) termed the *conductive model*, and (b) a model that additionally accounts for convective heat transport by computing the fluid flow in the melt pool induced by surface tension gradients, assuming negligible deformations of the free surface and that the melted fluid is incompressible, termed the *convective model*. Both models were stated in a frame moving with the laser (*xyz* in Fig. 1), in which the temperature distribution is expected to reach a steady state, facilitating their numerical solution. The three-dimensional melt pools from the computational models were transformed into two-dimensional cross-sections to compare against the sectioned samples by projecting all points that reached the liquidus temperature onto a plane behind the melt pool, see Fig. 1. The boundary of all projected points constitutes the computational 2D melt pool trace that we compare against the experimental one. Similarly, for any temperature *T*, all points that reached a temperature above *T* are projected onto the same plane. We refer to the boundary of the projection as the trace of the *T*-isotherm.

Material properties for the computational models were extracted from the literature, and assumed to be accurate enough for this work. We considered two parameters as unknown, the absorption coefficient $\alpha$ and sample surface location $z_S$, i.e., the location where the sample surface lies along the laser’s optical axis. The reason for this choice lies in the complexity of the laser absorption process and the power density distribution of the laser. It is difficult to predict the value of $\alpha$, and it is not uncommon to treat it as an unknown^29^. With $\alpha$ we accounted for all the channels of power loss, e.g. reflection of the laser and thermal radiation and convection to the surrounding atmosphere. Its value changes a little from experiment to experiment, because of the different power and scanning speed combinations. The motivation to treat the sample surface location as an unknown is twofold. First, we did not control the position of the top surface of the substrate along the optical axis well enough. Second, we shall see below that in some instances a conductive model can reproduce the 2D melt pool traces by selecting a value for $z_S$ that is inconsistent with the experiments, an important observation towards answering the question in this paper. Hence, while the usual practice in the literature is to assume the laser power density distribution at the beam waist (or the focal plane), we considered the possibility that the sample surface location $z_S$ did not coincide with laser focal plane. We deliberately did not assume the same value of $z_S$ for each track on a substrate’s surface, and examined the consistency of the values obtained from adjusting the computational models.

To determine the values of $\alpha$ and $z_S$, we sampled points in a region of the $\alpha$-$z_S$ space, computed the results of the models at each one of the points, and then selected those that defined 2D melt pool traces with the smallest error in each case. The error *e* was defined as the maximum distance among a set $B_1$ of sampling points in the computational curve to a set $B_2$ of sampling points in the experimental one, $e=\max _{p_1\in B_1}\min _{p_2\in B_2}|p_1-p_2|$.

## Results

### Both models can reproduce the 2D melt pool traces

**Figure 2:**
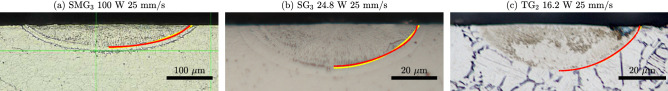
Comparison of experimental and computational 2D melt pool traces for 3 of the 11 cases in Table 1. Results of the convective model are shown in red, while those of the conductive model are in yellow. The optimal values for $\alpha$ and $z_S$ were used to obtain the computational 2D melt pool traces in each case, and are reported in Table 1. Only half of the computed trace is shown so that the experimental one is not obstructed.

By scanning the $\alpha$-$z_S$ space with either the conductive or the convective model, we found values that produce computational 2D melt pool traces close enough to the experimental results in each one of the 11 cases. Comparisons of the computational and experimental 2D melt pool traces are shown in Fig. 2 and in the Supplementary Information. We defined 1708 K as the liquidus temperature for 17-4PH SS, and 1986 K for Ti-6Al-4V. In all 17-4PH SS images two traces are clearly distinguishable, albeit with different intensity, since different etchants were utilized for samples in the SG and SMG groups (c.f. Methods). Since the region with marked feature refinement begins on the innermost trace, this was the one identified as the trace of the liquidus isotherm. The appearance of multiple traces around weld lines has also been observed in earlier works in 17-4PH SS^38^. Table 1 shows the error between the computational and experimental curves, which is always smaller for the convective model.

**Table 1:** Conditions and results for each one of the 11 experiments. Experiments within group SG or within group TG have been conducted on the surface of the same substrate, and thus should have very similar sample surface location $z_S$. The results here include the optimal values of $\alpha$ and $z_S$, i.e., those that minimize the error between the computational and experimental curves for each one of the two models. Two dimensional melt pool traces were deemed similar enough to the experimental one for errors below the threshold.

Experiment Label	${\hbox {SMG}}_1$	${\hbox {SMG}}_2$	${\hbox {SMG}}_3$	${\hbox {SG}}_1$	${\hbox {SG}}_2$	${\hbox {SG}}_3$	${\hbox {SG}}_4$	${\hbox {TG}}_1$	${\hbox {TG}}_2$	${\hbox {TG}}_3$	${\hbox {TG}}_4$
Beam type, material	Multi-Gaussian, 17-4PH SS			Ast. Gaussian, 17-4PH SS				Ast. Gaussian, Ti-6Al-4V			
Power ($\text {W}$)	100	100	100	16.2	16.2	24.8	24.8	16.2	16.2	11.6	11.6
Speed ($\text {mm/s}$)	6.25	12.5	25	25	50	25	50	100	25	25	12.5
$\alpha$–Conductive	0.595	0.590	0.670	0.302	0.318	0.274	0.288	0.395	0.407	0.435	0.435
$z_S$–Conductive (mm)	12.25	11.00	11.50	0.05	0.00	0.80	0.75	0.20	0.45	0.20	0.15
Error (*e*)–Conductive ($\upmu$m)	4.8	5.5	3.7	0.48	0.30	0.91	1.14	0.65	0.73	0.70	1.00
$\alpha$–Convective	0.450	0.460	0.555	0.315	0.327	0.306	0.331	0.420	0.438	0.448	0.448
$z_S$–Convective (mm)	6	6	8.5	$-$0.3	$-$0.3	$-$0.2	$-$0.4	$-$0.3	$-$0.3	$-$0.1	$-$0.1
Error (*e*)–Convective ($\upmu$m)	2.9	2.7	1.6	0.36	0.30	0.80	0.60	0.46	0.68	0.61	0.81
Error Threshold ($\upmu$m)	5	5	5	0.7	0.7	1	1	1	1	1	1

### Multiple 3D melt pools can match an experimental 2D melt pool trace

We show next that in addition to the optimal values of $\alpha$ and $z_S$ reported in Table 1, other combinations of values of $\alpha$ and $z_S$ lead to computational 2D melt pool traces that are compellingly similar to the experimental ones; the errors are almost as small. To this end, we sampled regions of the $(\alpha , z_S)$ space at regular intervals, and for each pair the resulting computational 2D melt pool trace was considered *similar* to the experimental one whenever the error fell below a threshold, indicated in Table 1. The thresholds were chosen (see Table 1) so that a visual inspection of the computational 2D melt pool traces could be reasonably said to match the experimental curves well. This resulted in threshold values that were at most a few percents of the average 2D melt pool trace depth in each group of experiments.

**Figure 3:**
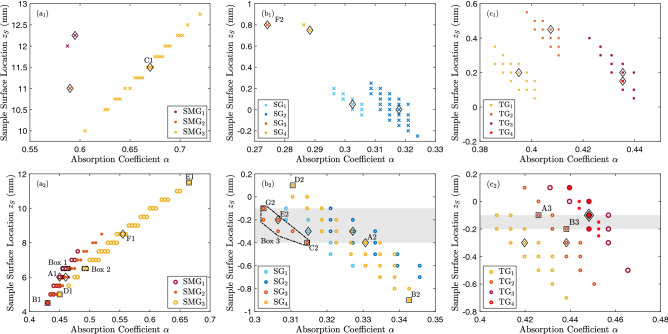
Pairs $(\alpha , z_S)$ that generate 2D melt pool traces with an error smaller than the error threshold in Table 1 for each experiment, out of a large set of sampled pairs. Top row: conductive model. Bottom row: convective model. The grayed region indicates the range of sample surface locations common to all experiments in the SG and TG groups. No common set of values was found with the conductive model, despite the fact that experiments in the SG and TG group were all performed at the same sample surface location. Larger error thresholds of 1.2 $\upmu$m instead of 1 $\upmu$m and 5.5 $\upmu$m instead of 5 $\upmu$m had to be selected for the ${\hbox {SG}}_4$ and ${\hbox {SMG}}_2$ cases, respectively, to find at least one pair with the conductive model. Some $(\alpha , z_S)$ values are labeled (e.g. B2) or boxed to be referenced elsewhere, and optimal values in Table 1 for each experiment are indicated with diamonds.

All ($\alpha ,z_S)$ pairs that led to computational 2D melt pool traces similar to experimental ones are shown in Fig.  3. The first notable observation is that there are in fact many pairs, particularly in the case of the convective model. These pairs correspond to different three-dimensional melt pools, as discussed below. The second notable observation is that in the convective model the values for $\alpha$ and $z_S$ for each experiment vary across a substantial range; values of $\alpha$ can vary between 5% to 20%, and the variation in values in $z_S$ correspond to substantial changes in the laser power density distribution; see Fig. 1c for the Gaussian beam, and Fig. 1d for the multi-Gaussian beam. In contrast, a much narrower band of variation is found in the conductive case. In particular, in the ${\hbox {SMG}}_2$ and ${\hbox {SG}}_4$ cases the error threshold had to be slightly increased to find at least one match with the conductive model. Regardless of the model, in all SG and TG cases the trend consistently shows that a narrower beam in the direction transversal to the laser path requires a higher fraction of the energy to be absorbed to obtain a similar 2D melt pool trace, while the opposite is true for the SMG case (c.f. Supplementary Information). This behavior can be intuitively explained for the axisymmetric beam in the SMG case, since the power density decreases with $z_S$ (c.f. Fig. 1); it is less intuitive for the astigmatic beam.

**Figure 4:**
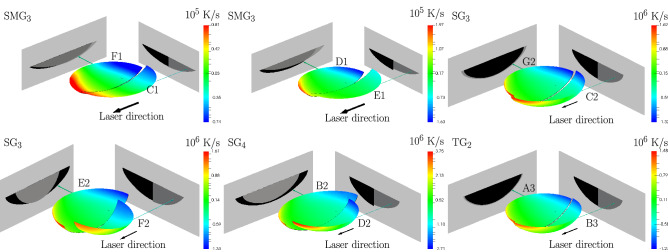
Comparison of melt pools computed by the models. The melt pools are projected onto planes perpendicular and parallel to the laser velocity. The contours are cooling rates $\dot{T}$. The left column shows comparisons between the convective and conductive models for the optimal ($\alpha$,$z_S$) pairs in Table 1 for the ${\hbox {SMG}}_3$ and ${\hbox {SG}}_3$ cases. The middle column shows a comparison between convective models with $(\alpha ,z_S)$ values at opposite extremes of the ranges for the ${\hbox {SMG}}_3$ and ${\hbox {SG}}_4$ cases. The right column shows a comparison between convective models with $(\alpha ,z_S)$ values at opposite extremes of the grayed regions for the ${\hbox {SG}}_3$ and ${\hbox {TG}}_2$ cases. In each case, the melt pool traces of the two melt pools are very close to the experimental results.

Melt pools that generate similar 2D melt pool traces can be substantially different, as illustrated in Fig. 4. The difference is particularly striking in the case of the astigmatic beam in ${\hbox {SG}}_4$, with two melt pools of significantly different length but similar width, both a result of the convective model with different values of $\alpha$ and $z_S$. This is a reflection of two melt pools generated with the longest axis of the elliptic power density distribution of the Gaussian beam oriented alongside and transversely to the beam direction. The differences are equally striking between the melt pools generated with the optimal values in Table 1 for the conductive and convective cases in ${\hbox {SG}}_3$ (bottom-left of Fig. 4); while the 2D melt pool trace is matched by both models, the melt pools are substantially different. The constrast between melt pools is less significant in the case of an axisymmetric beam, as shown by the milder differences in melt pool length for the ${\hbox {SMG}}_3$ case in the same figure.

### The range of convective 3D melt pool shapes can be narrowed by more careful control of the laser beam

A crucial observation that emerges from Fig. 3 is that if the value of $z_S$ is assumed to be *exactly known*, then for each experiment there is only a narrow range of values of $\alpha$ for which the computed 2D melt pool traces are similar to the experimentally measured ones. For example, setting $z_S=6.5~\text {mm}$ in Fig. 3a2 leads to values of $\alpha$ between 0.455 and 0.465 for ${\hbox {SMG}}_1$ (Box 1), or between 0.490 and 0.495 for ${\hbox {SMG}}_3$ (Box 2). More generally, if the value of $z_S$ is assumed to vary over a narrow range, then only a small region in the $(\alpha , z_S)$ plane would lead to computed 2D melt pool traces that are similar to experimentally measured ones, e.g. Box 3 for ${\hbox {SG}}_3$ in Fig. 3b2. Small regions in the $(\alpha , z_S)$ space have largely similar computed 3D melt pool shapes, as illustrated by the comparison in the right column in Fig. 4 of the 3D melt pool shapes for the most dissimilar $(\alpha , z_S)$ pairs for ${\hbox {SG}}_3$ and ${\hbox {TG}}_2$ within the grayed region in Fig. 3b2,c2. Thus, careful control of the power density distribution generated by the laser beam on the substrate’s surface would lead to a narrow range of computed 3D melt pool shapes, increasing the confidence in the computed thermal histories.

The precise location of the surface sample was not tightly controlled in our experiments; its value was $z_S\approx 5\pm 4$ mm for the SMG group, and $z_S\approx 0\pm 0.5$ mm for the SG and TG groups. However, in the SG and TG cases, a narrow range for $z_S$ can be obtained by using the fact that all experiments were conducted with a common sample surface location, and hence the value of $z_S$ should be common to all of them. The range of values of $z_S$ common to all experiments in each group is shown as the grayed area in Fig. 3b2,c2. Such range exists only for results of the *convective model*, and it is consistent with the aforementioned experimental values. This effectively defines a small region in the $(\alpha ,z_S)$ space for each experiment, and limits the range of 3D melt pool shapes.

### The results of the conductive model are inconsistent with the experimental settings

The lack of a common range of values of $z_S$ for all experiments in the SG and TG group, reflected by the absence of a grayed region in Fig. 3b1,c1, is in direct contradiction with the fact that all experiments (SG/TG) within the same group have been conducted on the same substrate surface without altering the surface location along the optical axis. The differences in the values of $z_S$ within each group are non-negligible, since as illustrated in Fig. 1c, a change of 0.5 mm in the value of $z_S$ leads to substantially different power density distributions. Inconsistencies are also found in experiments in the SMG group, since the computed values of $z_S$, shown in Fig. 3a1, fall outside the range $z_S\approx 5\pm 4~\text {mm}$, a generously defined range for the location of the surface during the experiments. Similarly, having a more accurate knowledge of $z_S$ would make it impossible to match the melt pool trace with the conductive model for some of the experiments in the SG and TG group; as we found in the SMG group.

Additional evidence that the conductive model is inconsistent with the experiments follows from the computed values of the absorption coefficient. Values of $\approx$30% for the SG group and of $\approx$40% for the TG group are in the range reported for a Gaussian beam on flat substrates for SS 316L and Ti-6Al-4V in^39^. In contrast, both models suggest very different ranges for $\alpha$ for the SMG group, $\approx 50$% for the convective model, and $\approx 60$% for the conductive one. While literature values of absorptance around 50% have been reported for some stainless steels^3,40^, we failed to find values above 60% in the absence of a keyhole.

### Thermal histories in the HAZ can be computed consistently with the convective model

The microstructure that forms in the solidified melt pool and surrounding heat affected zone (HAZ) depends heavily on the thermal histories of the material points. In particular, the cooling rate $\dot{T}$, the magnitude of the temperature gradient *G*, and the solidification front speed *R* play a crucial role on grain morphology and martensite formation. We examine whether thermal histories are similar among different pairs $(\alpha ,z_S)$ with 2D melt pool traces similar to the experimental ones.

**Figure 5:**
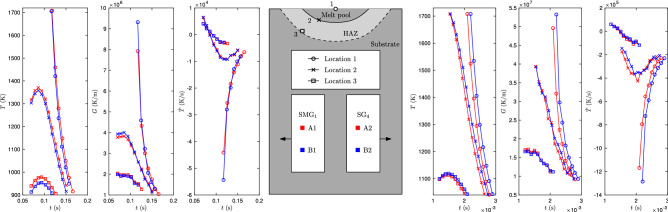
Time history of the temperature *T*, temperature gradient *G*, and cooling rate $\dot{T}$, for the ${\hbox {SMG}}_1$(left) and ${\hbox {SG}}_4$(right) cases. Shown are the histories at three different points at the locations sketched, as computed by the optimal (A1 and A2) and edge values (B1 and B2) of $(\alpha , z_S)$ for the convective model. Only the cooling down histories below the liquidus temperature are plotted.

Figure 5 shows the time-histories of the temperature *T*, *G*, and $\dot{T}$ for ${\hbox {SMG}}_1$ and ${\hbox {SG}}_4$ at 3 different points in the substrate: one at the top surface and center of the melt pool, another one on the melt pool trace, and a third one on the HAZ. Time histories were computed with both optimal values (A1 and A2) and “edge” values (B1 and B2) of $(\alpha ,z_S)$ for the convective model, as a way to evaluate how these histories might vary in the most extreme cases. Overall, the thermal histories at each one of the points are consistently computed; they are all very similar. Sample distributions of *G*, *R*, and *G*/*R* on the liquidus isosurface are shown in the Supplementary Information.

**Figure 6:**
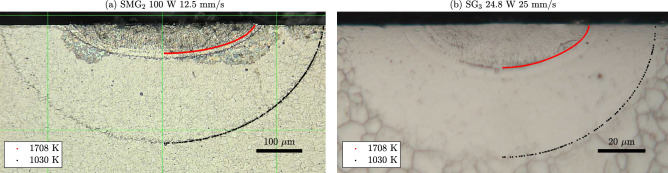
Trace of the boundary of the heat affected zones (HAZ), seen as a jagged line in ${\hbox {SMG}}_2$ and as the place where grain boundaries become faint in ${\hbox {SG}}_3$. The traces of two isotherms are shown computed with the optimal values of $(\alpha , z_S)$ and the convective model in each case. These include the liquidus isotherm (red) and the $1030~\text {K}$-isotherm (black). The HAZ boundary coincides with the trace of an isotherm in both cases.

A second test on the computation of thermal histories follows from the trace of the HAZ boundary revealed in the experiments on 17-4PH SS. Two of such zones are shown in Fig. 6 for ${\hbox {SMG}}_2$ and ${\hbox {SG}}_3$. Since the SMG and SG samples were etched with different etchants, the HAZ appears differently in each one. In the SG case the grain boundaries inside the HAZ region became markedly faint, nearly invisible, in stark contrast with the intact substrate material around it. Meanwhile, a jagged boundary between the HAZ and the intact region appears in the SMG case.

We found that the boundary of the HAZ may correspond to a 3D isotherm of the convective model. To see this, we computed the 2D traces of *T*-isotherms for *T* between $900~\text {K}$ and $1200~\text {K}$ in 5 K increments for all pairs $(\alpha , z_S)$ in Fig. 3a2,b2, and for each experiment determined the range of values of *T* for which the 2D traces of the isotherms were deemed to match the 2D trace of the HAZ boundary well (see Supplementary Information). The temperature ranges were 995–1045 $\text {K}$(${\hbox {SMG}}_1$), 1000–1060 $\text {K}$(${\hbox {SMG}}_2$), 1020–1085 $\text {K}$(${\hbox {SMG}}_3$), 1030–1040 $\text {K}$(${\hbox {SG}}_1$), 1040–1065 $\text {K}$(${\hbox {SG}}_2$), 990–1015 $\text {K}$(${\hbox {SG}}_3$) and 1065–1105 $\text {K}$(${\hbox {SG}}_4$). The similarity between the ranges strongly suggest that the HAZ boundary may be a 3D isotherm. The computed 2D traces of the $1030~\text {K}$-isotherms for the optimal values of $(\alpha , z_S)$ in each case are shown in Fig. 6, displaying a remarkable overlap with the 2D trace of the HAZ boundary. A comparison for the conductive model is reported in the Supplementary Information.

## Discussion

As hinted to in the introduction, the key observation that gives rise to the question we examine in this paper can be stated as follows. In the frame of the laser beam, the steady state temperature of the solid region of the substrate can be described by a nonlinear convection–diffusion equation, with the convection given by the velocity of the laser beam, c.f. (1), and the nonlinearities arising from the dependence of the thermal diffusivity on the temperature. Because the particles in the solid region of the substrate are not moving in the laboratory frame, the temperature field therein is defined purely by heat conduction. Then, if the laser beam does not shine any power outside the melt pool surface, the steady state temperature history of every point in the solid region of the substrate is uniquely defined by the 3D shape of the melt pool boundary. In particular, this implies that the solidification front speed *R* and the magnitude of the temperature gradient *G* at the melt pool boundary are also uniquely defined. This is a somewhat straightforward consequence of a general result for nonlinear convection–diffusion equations^41^. Of course, in most cases a fraction of the energy of the laser beam is deposited outside the melt pool; however, since solutions of these equations depend continuously on the heat source^41^, if such fraction is small, then thermal histories in the solid region of the substrate arising from small but differing fractions of energy deposited outside the melt pool will be close.

Back to the question we examine in this paper, whether it is possible to recover the 3D shape of a melt pool with a model that reproduces the 2D melt pool trace, the results in Figs.  3 and 4 show that there exist many melt pools that have the same experimental 2D melt pool trace, with both the convective and the conductive model. Such melt pools can have substantially different (3D) shapes in the case of an astigmatic laser beam, but are quite similar in the case of an axisymmetric one among the experiments here. Analytically, it is possible to construct different beams that results in the same 2D melt pool trace (c.f. Supplementary Information). These facts show that inferring the 3D melt pool shape from a model that matches the 2D melt pool trace requires knowledge of the beam characteristics.

Estimating the 3D melt pool shape from a single experiment is clearly not possible, as illustrated in Fig. 4, unless tightened controls on the power density distribution on the surface are implemented; in this case, through better control of the sample surface location. In particular, in the case of the convective model, the range of melt pool shapes that match the 2D melt pool trace for each individual experiment is quite large, see Fig. 3. Nevertheless, through multiple experiments, we were able to narrow it down, as the grayed region in Fig. 3 indicates.

Even if the power density distribution of the beam on the substrate surface were perfectly known, the precise value of the absorption coefficient would still be unknown. The question here is if, given a precise knowledge of the power density distribution, there is at most a unique value of $\alpha$ whose 3D melt pool shape reproduces a given 2D melt pool trace. Since a smaller value of $\alpha$ indicates less power absorbed by the substrate, it is reasonable to think that 3D melt pools corresponding to larger values of $\alpha$ would contain the 3D melt pools generated by smaller values. This is certainly the case for a linear conductive model on the substrate. It is difficult to assert this in the presence of convection of the melt, and its veracity may depend on the way the surface tension depends on the temperature, so it is a non-trivial question. The narrow band that the values of $\alpha$ form for each experiment in Fig. 3 is consistent with an affirmative answer to this question, and hence supports the conclusion that by tightly controlling the power density distribution on the surface, it is possible to recover the 3D melt pool shape from the 2D melt pool trace.

Finally, the results suggest that we cannot ignore all convective effects in modeling any of the experiments herein, even though all melt pool shapes resemble sections of ellipsoidal shells typically associated with melt pools in predominantly conduction mode^42^. Even if we ignored the fact that the power density distribution needed to match the 2D melt pool trace is inconsistent with the experimental settings, as found here, a conductive model that matches the 2D melt pool trace does not necessarily approximate the 3D melt pool shape, or the isotherms in the HAZ (c.f. Supplementary Information). We expect a similar observation to be true for the more elongated melt pools that could appear at moderately larger laser beam speeds and powers.

On a different note, a hypothesis that emerges from Fig.  6 is that the HAZ boundary coincides with a 3D isotherm. We hypothesize that the experimentally-observed 2D trace of the HAZ boundary is outlining the region of the substrate that transformed from martensite to austenite at some point during the process; so the HAZ boundary is (close to) the ${\hbox {Ac}}_{1}$-isotherm. In three dilatometry tests of 17-4PH SS^43–45^, the austenite-martensite transformation takes place between 1073–1198 $\text {K}$, 888–1053 $\text {K}$, and 890–1023 $\text {K}$. An earlier study suggested a range of 1070–1200 $\text {K}$^38^. All of these ranges overlap with the range found in the results section, 990–1105 $\text {K}$, supporting the hypothesis of the nature of the observed HAZ boundary.

## Methods

### Experimental methods

Commercial grade 17-4PH SS and Ti-6Al-4V flat plates (thickness: SMG $5.972~\text {mm}$, SG $13.168~\text {mm}$, TG $14.966~\text {mm}$) were used in the experiments. Sample surfaces were sanded under cooling water. For the optical microscopy preparation, the samples were sectioned with coolant, mechanically polished, and etched with the following etching procedures: (a) samples in the SMG group were electro-etched for 45 s with $10\%$ Oxalic Acid, (b) samples in the SG group were etched with a modified glyceregia etchant, (c) samples in the TG group sample were etched with Kroll’s reagent. For experiments conducted on the surface of the same sample (in the SG and TG groups), at least 5 minutes passed between consecutive experiments, for the temperature of the sample to be back at room temperature. All experiments in the SMG and SG group were performed at least twice, with repeatable results.

### Computational models

The *convective* model is described by the following set of linear momentum and energy balance equations in a frame attached to the laser beam, indicated by Cartesian coordinates *xyz*, moving at a constant velocity ${\varvec{c}}$ with respect to the laboratory (see Fig. 1a). In a parallelepiped domain $\Omega$ fixed in this frame (G: $2~\text {mm}\times 1~\text {mm}\times 4~\text {mm}$, MG: $20~\text {mm}\times 10~\text {mm}\times 35~\text {mm}$ (width$\times$height$\times$length)), we seek a temperature field *T*(*x*, *y*, *z*, *t*), a pressure field *p*(*x*, *y*, *z*, *t*), and a velocity field *in the laboratory frame* ${{\varvec{v}}}(x,y,z,t)$, that satisfy that for all $t>0$ and all $(x,y,z)\in \Omega$,

1 $$\begin{aligned} \rho _0C(T)\Big (\frac{\partial T}{\partial t}+({\varvec{v}}-{\varvec{c}})\cdot \nabla T\Big )&=\nabla \cdot (k(T) \nabla T) \end{aligned}$$

2 $$\begin{aligned} \rho _0\Big (\frac{\partial {\varvec{v}}}{\partial t}+({\varvec{v}}-{\varvec{c}})\cdot \nabla {\varvec{v}}\Big )&=\nabla \cdot \varvec{\sigma } \end{aligned}$$

3 $$\begin{aligned} \nabla \cdot {\varvec{v}}&=0 \end{aligned}$$

4 $$\begin{aligned} \varvec{\sigma }&=\mu (T)(\nabla {\varvec{v}}+\nabla ^T {\varvec{v}})-p {\mathbb {I}}. \end{aligned}$$

Here *C*(*T*) is the specific heat, *k*(*T*) is the thermal conductivity, and $\mu (T)$ is the viscosity of the melt, all as a function of the temperature. The deformation of the solid, unmelted region, is modeled as a fluid as well, with a very large viscosity ($10^4$ the the viscosity of the fluid at 1708K for 17-4PH SS and 1973K for Ti-6Al-4V), which we obtain by extending the viscosity of the melt to lower temperatures. Both the fluid and the solid region are modeled as incompressible Newtonian fluids, a fact expressed by the constitutive relation for the stress field $\varvec{\sigma }$, (4), and the incompressibility constraint in (3). We ignored the specific volume change with temperature, and turbulence or friction in the mushy zone, as sometimes included in the literature^29^, as well as heat losses due to radiation and convection to the surrounding ambient gas (values estimated from the modeling results indicate these are at least two orders of magnitude smaller than the laser power). Additionally, $\rho _0$ is the mass density of the fluid metal, and ${\mathbb {I}}$ is the identity tensor in ${\mathbb {R}}^{3\times 3}$. The boundary conditions for both the temperature and the velocity fields are as follows. The upwind surface of the block (ahead of the laser) is assumed to be at room temperature, $300~\text {K}$, while the remaining surfaces (except the top) are assumed to be adiabatic. The velocity is set to be zero on all surfaces except the top. The boundary conditions on the top surface are that for all $t>0$ and all points on the surface

5 $$\begin{aligned} \big (k(T)\nabla T\big )\cdot {\varvec{n}}&=\alpha PI_{z_S} \end{aligned}$$

6 $$\begin{aligned} ({\mathbb {I}}-{\varvec{n}} \otimes {\varvec{n}}) \varvec{\sigma }\cdot {\varvec{n}}&=f_L(T)\frac{\partial \gamma (T)}{\partial T}({\mathbb {I}}-{\varvec{n}} \otimes {\varvec{n}})\nabla T \end{aligned}$$

7 $$\begin{aligned} {\varvec{v}}\cdot {\varvec{n}}&= 0. \end{aligned}$$

8 $$\begin{aligned} \gamma (T)&=\gamma _0-A(T-T_0)-C_1 T\ln \Big (1+C_2e^{\frac{C_3}{T}}\Big ) \end{aligned}$$

Here ${\varvec{n}}$ is the unit external normal, (5) expresses the influx of energy by the laser, where *P* is the total power and $I_{z_S}$ is the normalized power density distribution, (6) expresses that Marangoni forces impose a shear stress on the top surface of the fluid, while (7) states that the flow is confined to be parallel to the top surface of the substrate. The temperature-dependence of the surface tension $\gamma$ is expressed by (8), and $f_L:\rightarrow [0,1]$ represents a monotone transition from solid to fluid; its actual form is not very important because the transition region in which $f_L\not \in \{0,1\}$ is very narrow.

The *conductive* model follows by setting the velocity field ${\varvec{v}}$ to be identically zero everywhere in the domain, solving only (1) and (5) for *T*(*x*, *y*, *z*, *t*). In both models, we seek the steady state solution, in which $\frac{\partial T}{\partial t}=0$ and $\frac{\partial {\varvec{v}}}{\partial t}=0$.

The astigmatic Gaussian ($I_{z_S}^\text {G}$) and the multi-Gaussian ($I_{z_S}^\text {MG}$) normalized power density distributions have the form,

9 $$\begin{aligned} I_{z_S}^{\text {G}}(x,y)= \frac{2}{\pi }\frac{e^{-2\left[ \frac{x^2}{w_x(z_S)^2}+\frac{y^2}{w_y(z_S)^2}\right] }}{w_x(z_S)w_y(z_S)},\qquad \qquad I_{z_S}^{\text {MG}}(x,y)=\frac{2}{\pi } \frac{\sum _{n=-N}^{n=N} \sum _{m=-M(n)}^{m=M(n)} e^{-2\left[ \left( \frac{x-n\Delta }{w(z_S)}\right) ^2+\left( \frac{y-m\Delta }{w(z_S)}\right) ^2\right] }}{ \sum _{n=-N}^{n=N} \sum _{m=-M(n)}^{m=M(n)}w(z_S)^2} \end{aligned}$$

where $w_x(z_S)=w_{0,\text{ G }}\sqrt{1+(z_S-d_0)^2/z_{0,\text{ G }}^2}$, $w_y(z_S)=w_{0,\text{ G }}\sqrt{1+(z_S+d_0)^2/z_{0,\text{ G }}^2}$, $z_{0,\text{ G }}=\pi w_{0,\text{ G }}^2/\lambda$, $M(n)=\lfloor \sqrt{N^2-n^2}\rfloor$, $w(z_S)=w_{0,\text {MG}}\sqrt{1+(z_S/z_{0,\text {MG}})^2}$ and $z_{0,\text {MG}}=\pi w_{0,\text {MG}}^2/\lambda$. We set $N=18$, $d_0=1.487157939~\text {mm}$, $w_{0,MG}=10.5~\upmu \text {m}$, $\Delta =10~\upmu \text {m}$, $\lambda =1070~\text {nm}$, and $z_{0,G}=1.3625345~\text {mm}$. The reason the multi-Gaussian beam is modeled as a superposition of small Gaussian beams is simply that this model reproduced the beam power density distribution we measured, see Fig. 1d. We quantified the error introduced when approximating the experimental measurements to the analytical expression of the astigmatic Gaussian beam. At locations within 1 mm of the beam waist, the error in the length of the principal *x*-axis of the ellipse was between $-0.2\%$ and $7.7\%$, while for the *y*-axis was between $3.4\%$ and $8.6\%$.

Finite element discretizations were adopted to solve both models. The fluid problem is solved with a stabilized velocity and pressure formulation^46^, in which both the velocity and pressure fields are approximated with $P^1$-tetrahedra, and so is the temperature field. A semi-implicit integration scheme is used for both (1) and (2), in which the convection velocity is treated explicitly using the last time step’s value. Since it was very difficult to directly obtain the coupled, steady state solution in the process of solving the system, after an initial guess step assuming ${\varvec{v}}=0$, the transient energy and momentum equations were solved in alternative steps until the value of $\partial T/\partial t$ became negligible. The trace of a *T*-isotherm was obtained by sampling the *T*-isotherm, projecting the points as in Fig. 1, and computing the boundary of the resulting region with 2–20 $\upmu$m Alpha shapes. All results shown did not significantly change upon refinement of the finite element mesh. All computations were performed with the authors’ in-house computational code, and ran in up to 96 CPU cores. To converge to the steady state, the conductive model required only one step computed in a few seconds, while about ten minutes were needed for the convective model.

The properties applied in the computational model are temperature dependent. The properties for 17-4PH SS are from the IDS model^47^ and those for Ti-6Al-4V are from the software JMatPro Version 9.0 DEMO^48^. Properties beyond the available temperature range were kept constant and equal to their value at the highest temperature for which they were available. We based the model of the surface tension for 17-4PH SS on McNallan^49^ and Belton^50^. We set $A=4.3\times 10^{-4} \text {N}/(\text {m}\cdot \text {K})$, the value reported for the Fe-S or Fe-O system^49^. The remaining values are small perturbations of the those for the Fe-S system, $C_1=9.7274\times 10^{-5}~\text {N}/(\text {m}\cdot \text {K})$, $C_2=1.8317\times 10^{-5}$, and $C_3= 1.8991\times 10^{4}~\text {K}$. The final values are close to reported ones^51^.

## Supplementary Information

Supplementary Information.
