# Supplementary material for: A critical look at the prediction of the temperature field around a laser-induced melt pool on metallic substrates
Source: Sci Rep. 2021 Jun 9;11:12224. doi: 10.1038/s41598-021-91039-z (PMC8190039; doi:10.1038/s41598-021-91039-z)
Supplement: Supplementary file 1 — Supplementary Information. [file 41598_2021_91039_MOESM1_ESM.pdf]

Supplementary Information to
A Critical Look at the Prediction of the Temperature Field Around a Laser-Induced Melt Pool on Metallic Substrates

Yi Shu[1], Daniel Galles[2], Ottman A. Tertuliano[1], Brandon A. McWilliams[3], Nancy Yang[4], Wei Cai[1], and Adrian J. Lew[1,*]

[1]Stanford University, Department of Mechanical Engineering, Stanford, CA 94305, USA
[2]Oak Ridge Associated Universities, Oak Ridge, TN 37830, USA
[3]DEVCOM Army Research Laboratory, Aberdeen Proving Ground, MD 21005, USA
[4]Sandia National Laboratories, Livermore, CA 94550, USA
[*]lewa@stanford.edu

1 Laser power density distributions

We characterized the laser power density distributions of the laser beams. The measurements were performed with the Coherent PowerMax-Pro USB/RS Sensors for the multi-Gaussian beam and with the FLIR grasshopper 3 (GS3-U3-50S5C-C) for the astigmatic Gaussian beam. The astigmatic Gaussian beam (G) has a nearly axisymmetric power density distribution only when the sample surface is near the beam waist. The multi-Gaussian beam (MG) is nearly axisymmetric all along its optical axis. It is evident from Figs. S.1 and S.2 that the power density distributions of both beams cannot be represented by an axisymmetric Gaussian beam.

To provide a sense of the beam size, we defined a spot size for these non-Gaussian beams as follows. For the multi-Gaussian beam, it is the diameter of a circle in whose interior 86% of the power is shone; their values are listed in Table S.1. For the astigmatic Gaussian beam, the iso-intensity lines are self-similar ellipses, so the spot size was defined as the principal axis that is transversal to the laser motion of the ellipse in whose interior 86% of the power is shone; their values are listed in Table S.2.

$\lvert z_S \rvert$ (mm)	Spot Size (μm)	Power Fraction (%)
0	333	86.4
1	340	86.4
2	363	86.4
3	395	86.6
4	435	86.4
5	478	86.4
6	526	86.5
7	576	86.4
8	627	86.4

Table S.1. Spot size of the multi-Gaussian laser beam as a function of $|z_S|$, computed from the curves in red in Fig. S.2. The iso-intensity lines of the multi-Gaussian beam are nearly circular. The spot size is defined as the diameter of the circular region in whose interior 86.4% of the power is shone. The spot size increases as z_S moves away from the beam waist at $z_S \approx 0$ mm.

In Fig. 3(a1) in the manuscript the conductive model for the SMG group suggested a range $[10, 12.75]$ mm for the sample surface locations z_S, while in Fig. 3(a2) in the manuscript the convective model suggested a range $[4.5, 11.5]$ mm. Figure S.3 compares the power density distribution of the multi-Gaussian beam at $z_S = 6$ mm and $z_S = 12$ mm. The beam characterization experiments measured the power density distribution up to $z_S = 5$ mm from the beam waist, so both curves in Fig. S.3 are extrapolated by the model.

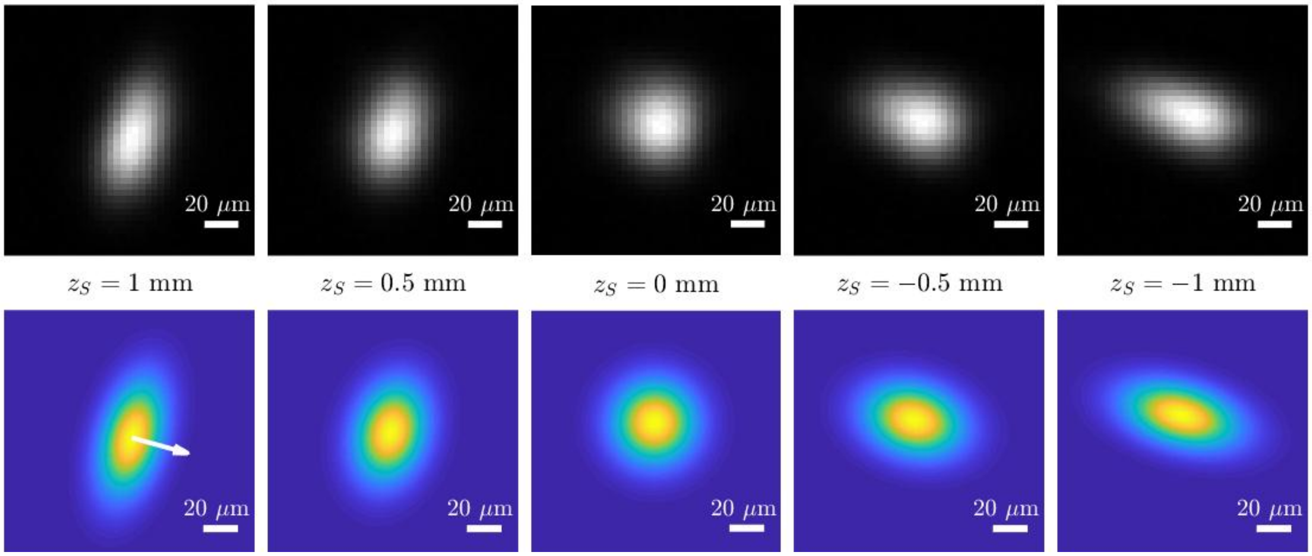

Figure S.1. Top: Measured power density distribution for the astigmatic Gaussian beam at different optical axis locations; lighter colors correspond to higher power density values. Bottom: Analytic representation of the power density distributions. The arrow indicates the laser scanning direction. The laser motion direction is aligned with one of the principal axes of the ellipse.

z_S (mm)	Section Spot Size (μm)	Axis Ratio
-1.00	45.76	0.51
-0.90	46.91	0.54
-0.80	48.25	0.57
-0.70	49.76	0.61
-0.60	51.41	0.65
-0.50	53.20	0.70
-0.40	55.12	0.75
-0.30	57.14	0.80
-0.20	59.27	0.86
-0.10	61.48	0.93
0.00	63.78	1.00
0.10	66.14	1.08
0.20	68.57	1.16
0.30	71.06	1.24
0.40	73.60	1.34
0.50	76.19	1.43
0.60	78.82	1.53
0.70	81.48	1.64
0.80	84.18	1.74
0.90	86.91	1.85
1.00	89.67	1.96

Table S.2. Spot size and axis ratio of the astigmatic Gaussian beam as a function of z_S. The iso-intensity lines of the power density distribution are ellipses whose principal axis are parallel (axis 1) and transversal (axis 2) to the direction of motion of the laser. The ellipse in whose interior 86.4% of the power of the laser falls is used to define the values listed in the table, in which the length of axis 2 is the spot size, and the length of axis 2 over the length of axis 1 is defined as the axis ratio.

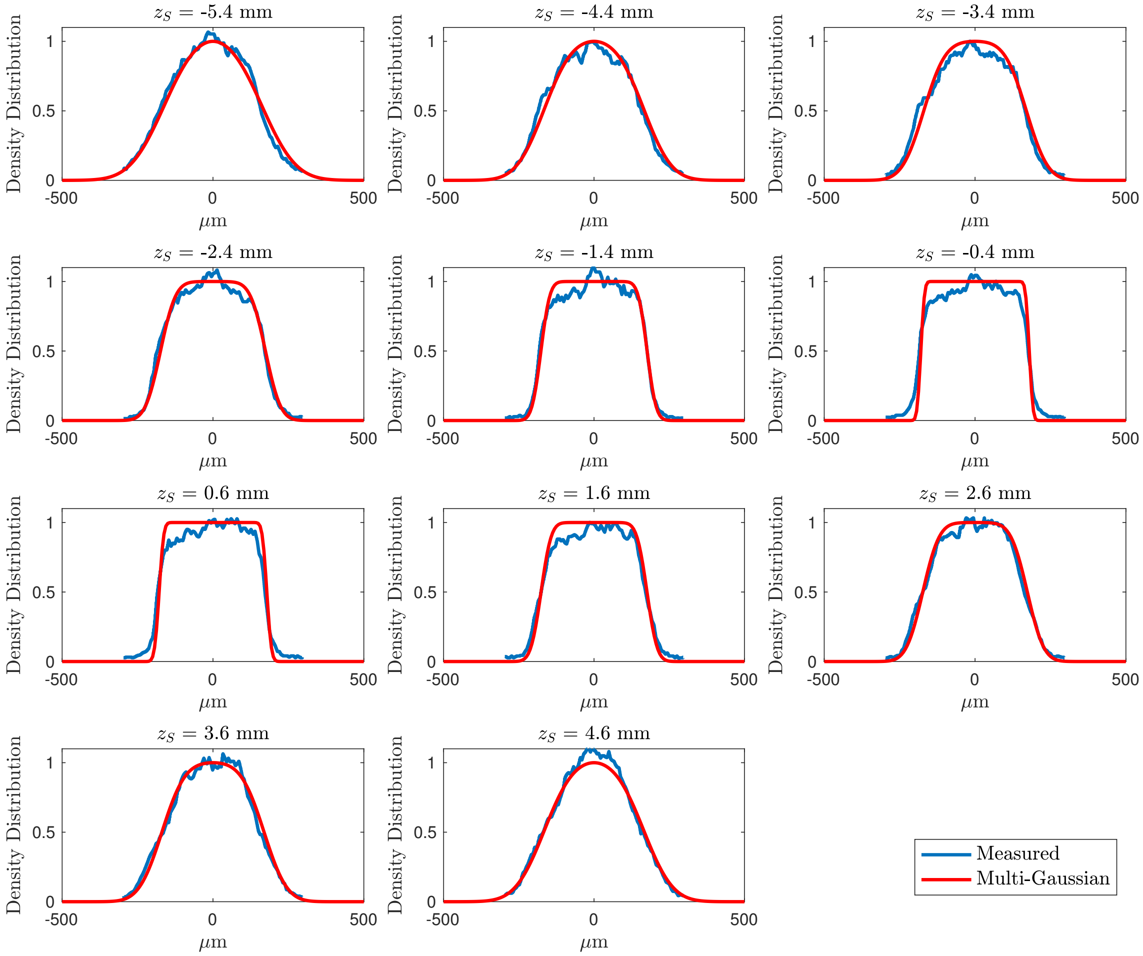

Figure S.2. Measured power density distribution of the beam in the SMG group (blue) and its multi-Gaussian model (red) for different sample surface locations z_S. This beam is almost axisymmetric, so only a section of the beam along the diameter is shown. The power density distribution is close to a top-hat shape near the beam waist and Gaussian far from the beam waist. The vertical axis shows a normalized intensity for the analytic representation, with different normalization factors for each value of z_S.

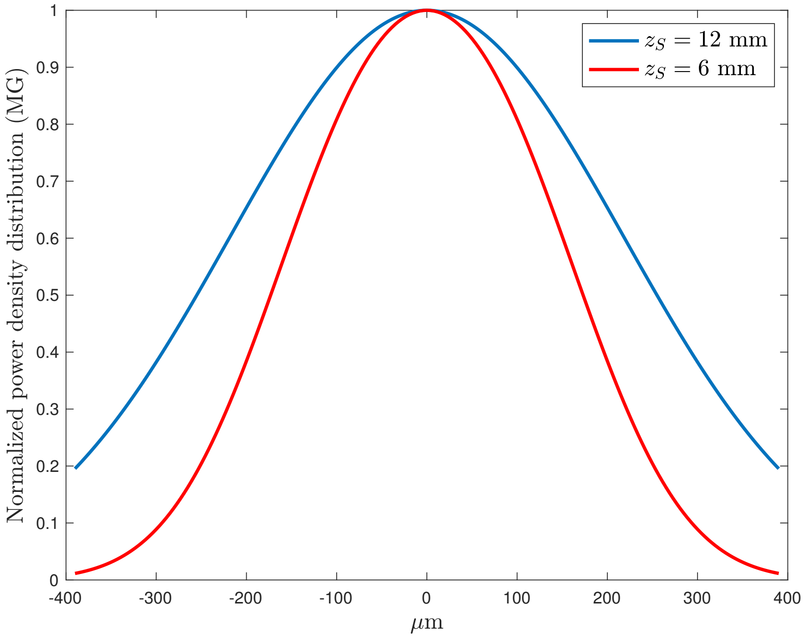

Figure S.3. Section plot of the power density distributions of the multi-Gaussian beam when $z_S = 6$ mm and $z_S = 12$ mm. The differences of the power density distribution cannot be neglected.

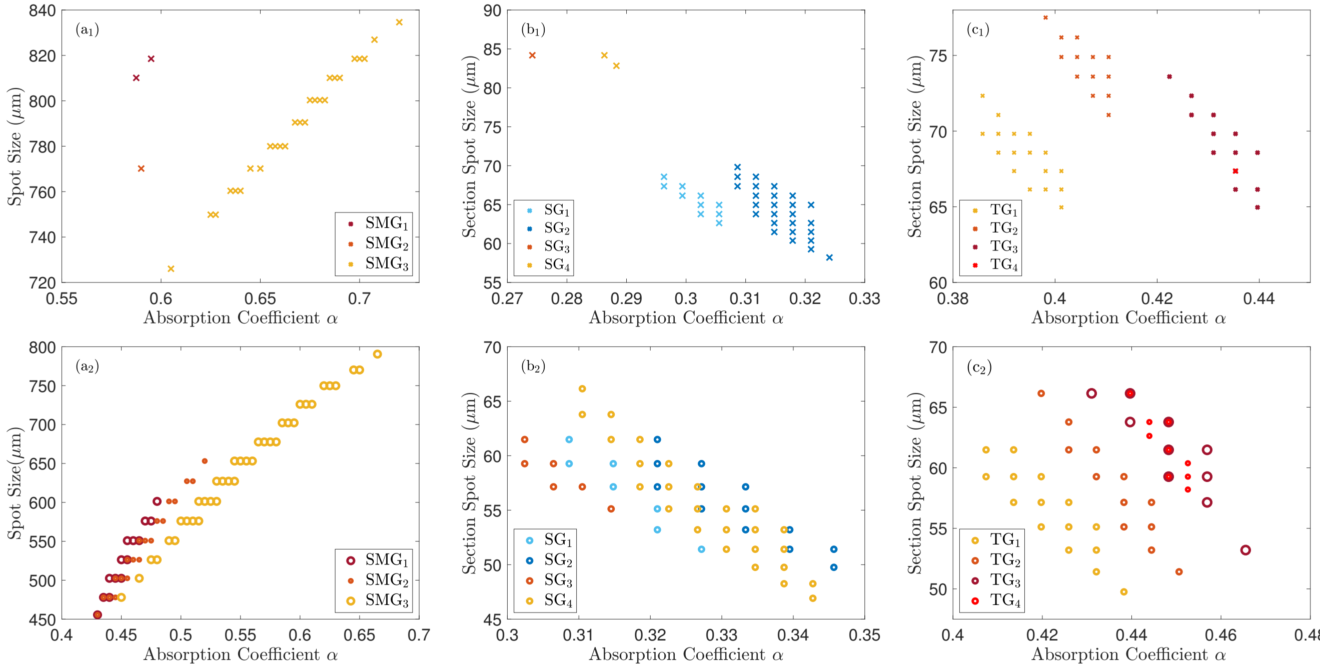

Figure S.4. Pairs of α and spot sizes that generate 2D melt pool traces with an error smaller than the error threshold in Table 1 in the manuscript for each experiment, out of a large set of sampled pairs. Top row: conductive model. Bottom row: convective model. The maps from z_S to a spot size value are described in Tables S.1 and S.2. The spot size is a measure of the maximum width of the laser beam in the direction transversal to the laser scanning direction, as defined in §1.

2 Complete comparison of computational and experimental melt pool traces

We conducted 11 experiments in three groups SMG, SG and TG and only one result of each group is shown in the manuscript. The complete set of comparisons across all experiments between the experimental and computational 2D melt pool traces, for both the convective and conductive models, are shown in Figs. S.5, S.6, and S.7.

To support a statement in the manuscript we also include Fig. S.4, which shows the data in Fig. 3 in the manuscript plotted in terms of the spot size instead of the sample surface location z_S. This figure shows that for the SG and TG groups, a narrower beam in the direction transversal to the laser path requires a higher fraction of the energy to be absorbed to obtain a similar 2D melt pool trace, while the opposite is true for the SMG case.

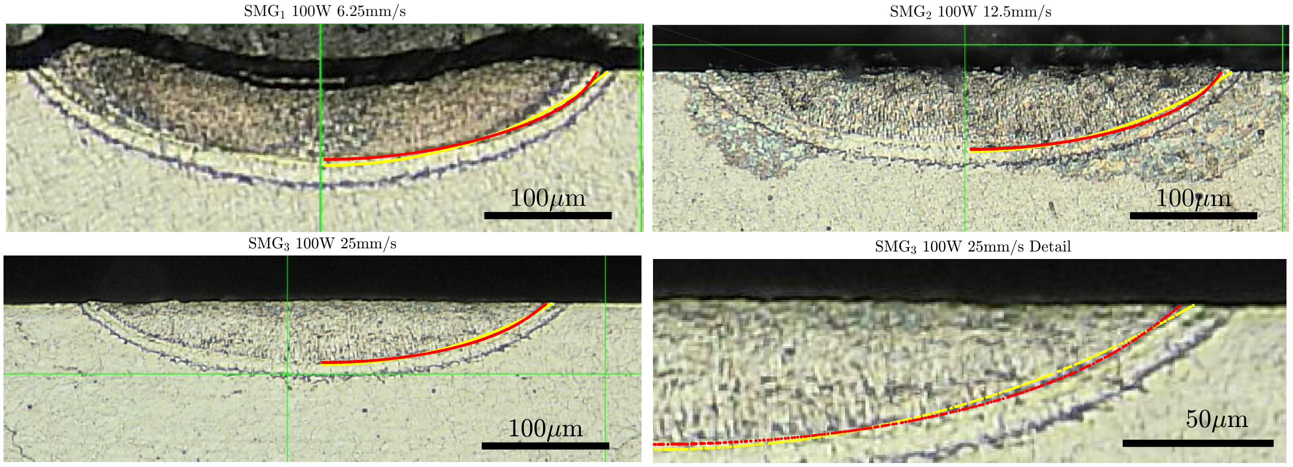

Figure S.5. Comparison of experimental and computational 2D melt pool traces on 17-4PH SS with a multi-Gaussian laser beam. Results of the convective model are shown in red, while those of the conductive model are in yellow. The optimal values for α and z_S used to obtain the computational melt pool traces in each case are reported in Table 1 of the manuscript. The bottom-right figure is an enlarged view of the bottom-left one.

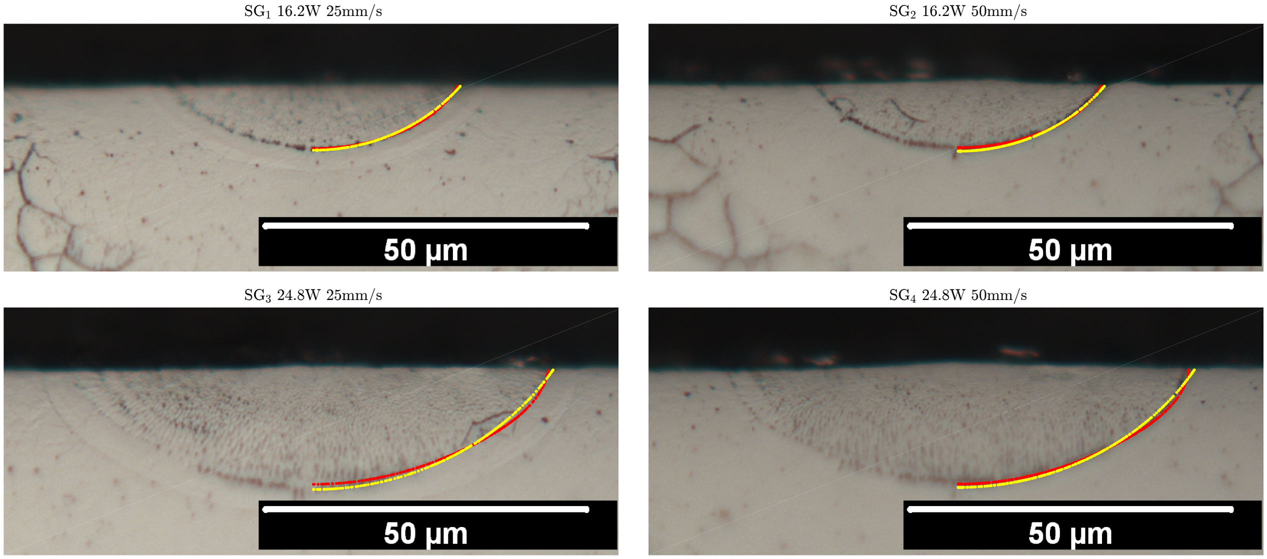

Figure S.6. Comparison of experimental and computational melt pool traces on 17-4PH SS with an astigmatic Gaussian laser beam. Results of the convective model are shown in red, while those of the conductive model are in yellow. The optimal values for α and z_s used to obtain the computational melt pool traces in each case are reported in Table 1 of the manuscript.

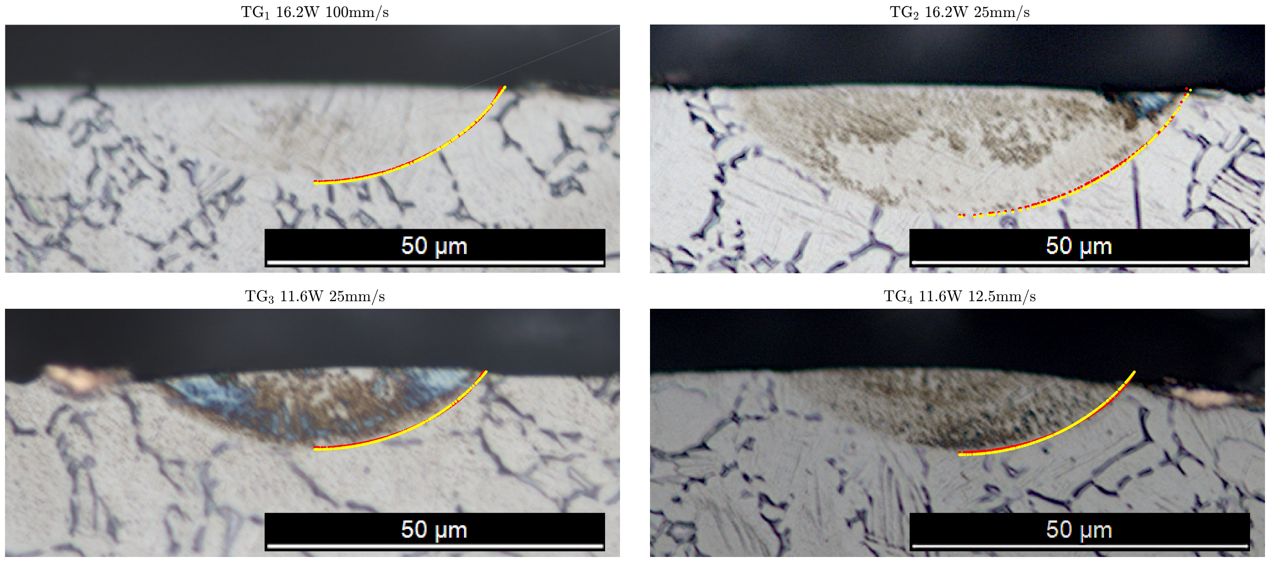

Figure S.7. Comparison of experimental and computational melt pool traces on Ti-6Al-4V with a astigmatic Gaussian laser beam. Results of the convective model are shown in red, while those of the conductive model are in yellow. The optimal values for α and z_s used to obtain the computational melt pool traces in each case are reported in Table 1 of the manuscript.

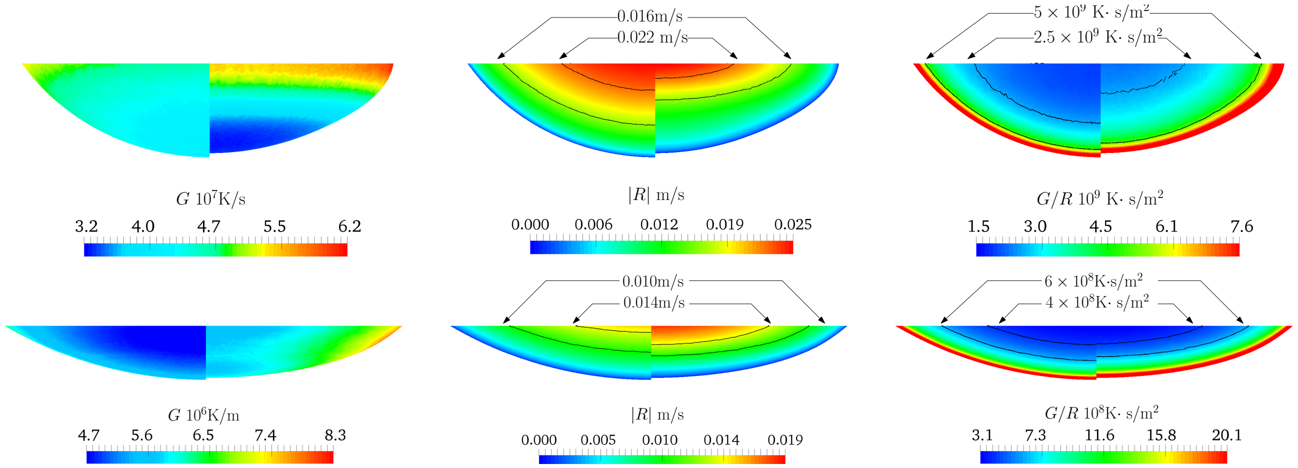

Figure S.8. Thermal gradient G, solidification velocity R, and G/R for the best values of (α, z_S) for the SG$_3$ (E2 and F2, Top) and SMG$_3$ (F1 and C1, bottom) cases. In each figure, contour plots from the conductive (left) and convective (right) model are shown. The values for these plots are obtained from projecting onto the back plane the values of G and R on the solidifying part of the liquidus isotherm, as illustrated in the introduction of the manuscript. Since $R = 0$ on the widest part of the melt pool, G/R is singular on the melt pool trace.

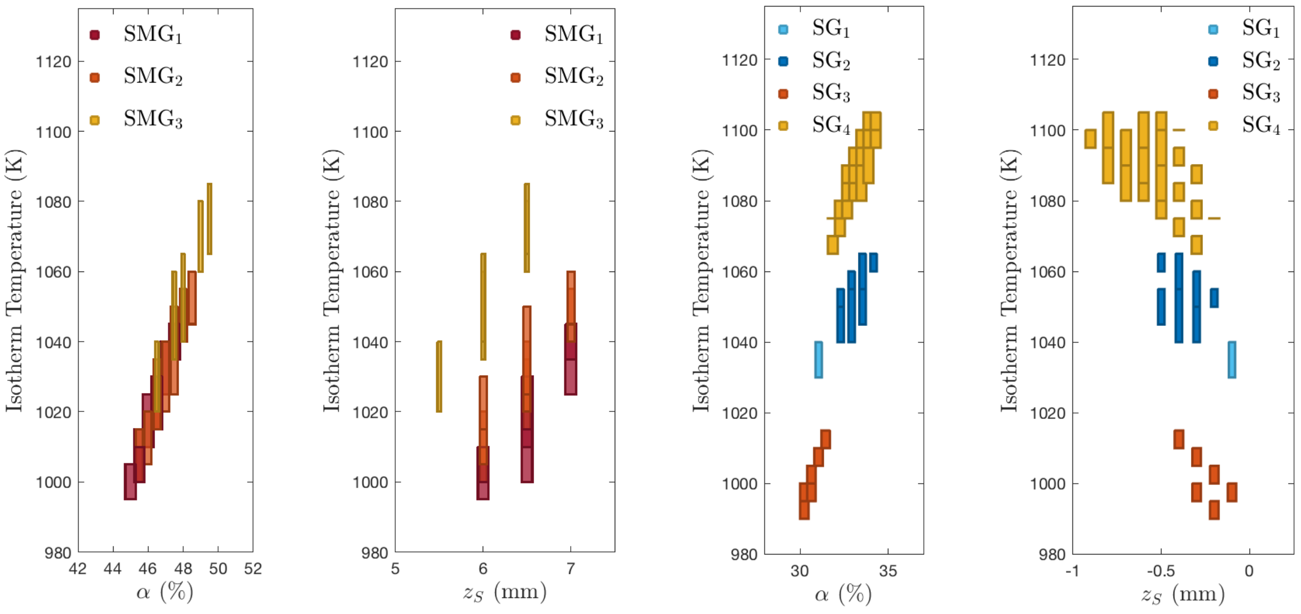

Figure S.9. Computed temperature range for which isotherms approximate the experimental HAZ boundary for the SG and SMG groups, for each pair (α, z_S) for which the trace of the computed melt pool matches the experimental one (those in Fig. 3 in the manuscript). The dependence of α and z_S are depicted in separate plots. Only HAZ boundaries computed with the convective model are close enough to the experimentally measured one, e.g. Fig. S.10.

3 Comparison of G, R and G/R between models

A comparison of the values of G, R and G/R at the solidification front computed by the optimal (α, z_S) pair for each model is shown in Fig. S.8 for the cases SG$_3$ and SMG$_3$. The values of G, R, and G/R are typically adopted to evaluate whether columnar or equiaxed grains will form. These contours complement the melt pool shapes and cooling rates for the same values of (α, z_S) in Fig. 4 in the manuscript. While the range of values for any of the three variables in the two models is within the same order of magnitude, their distribution in the melt pool boundaries is not. This is primarily a consequence of the convective currents that develop inside the melt pool. As sketched in the introduction, the flow on the surface of the melt pool moves from the center to the boundaries, carrying with it hot fluid that increases the value of G near the top end point of the melt pool trace, as shown in Fig. S.8. A simplified conclusion of this observation is that the same, colder fluid then moves to the bottom of the melt pool, leading to the lower values of G observed in the same figure. The contours of G in the SMG, convective case, signal that a more complex behavior is taking place. Corresponding differences are observed in the values of R; however, the values of G/R are quite similar in both models.

4 Isotherms, phase transformation and the HAZ Boundary

The trace of a T-isotherm is deemed to match the boundary of the HAZ well if it approximates the width and depth of the HAZ boundary with relative errors that range between about 3% and 8%, with the range selected for each case so that matches appear visually close to the experimental curve. For each pair (α, z_S), the range of values of T for which such match is found is shown as a vertical bar in Fig. S.9. The computed 1030K-isotherms for the optimal values of (α, z_S) in SMG$_2$ and SG$_3$ are shown in Fig. S.10, displaying a remarkable overlap with the HAZ boundary (the curves for the convective model appear in Fig. 6 in the manuscript as well, and are reproduced here for comparison).

A last remark is that not all the pairs (α, z_S) identified as matching the trace of the melt pool have the HAZ boudary as an isotherm. One such example is the set of optimal values for the convective model in SMG$_3$, $(0.555, 8.5\text{mm})$, which despite of minimizing the error in matching the melt pool trace, has not been included in Fig. S.9.

An interesting observation about the conductive model arises from a similar analysis. No computed isotherm for any of the optimal values of (α, z_S) in each case of the SMG group was close enough to the 2D trace of the HAZ boundary, as illustrated by the blue curves in Fig. S.10(a); these are the closest 2D traces of isotherms to the 2D trace of the HAZ boundary. Therefore, even though the 2D trace of the melt pool is matched well, it is apparent that the temperature field in the HAZ is incorrectly

computed. A closer looks reveals that the wider (and inconsistent with experimental conditions) beam that was necessary to match the 2D melt pool trace in this case deposits a large fraction of the energy of the laser outside the melt pool, distorting in this way the isotherms in the HAZ. This is illustrated in Fig. S.10 through the profile of the power density distribution that correspond to the (α, z_S) pair adopted in each case.

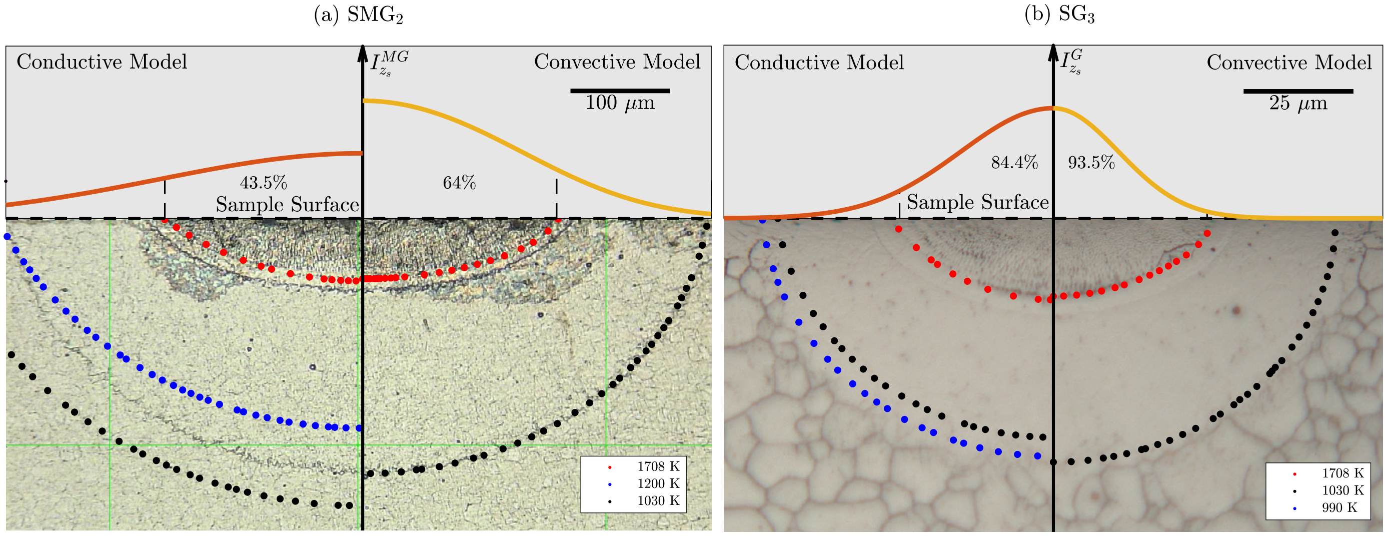

Figure S.10. Boundaries of the heat affected zones (HAZ), seen as a jagged line in SMG_2 and as the place where grain boundaries become faint in SG_3. The traces of several isotherms are shown, for both the conductive (left half) and convective (right half) models, computed with the optimal values of (α, z_S) in each case. These include the liquidus isotherm (red), the 1030K-isotherm (black), and the best matching isotherm to the HAZ boundary for the conductive model (blue). The power density distribution that generates them are depicted above the substrate. The HAZ boundary coincides with the trace of an isotherm of the convective model in both figures. However, it does not coincide with any isotherm of the conductive model for SMG_2. The culprit is the wider power density distribution extending beyond the HAZ boundary, necessary to reproduce the trace of the melt pool.

5 The same 2D melt pool traces do not indicate the same 3D melt pool shape

Analytically, it is possible to construct different beams that result in the same 2D melt pool trace. Concretely, consider the Rosenthal solution[1] to a pointwise beam traveling at a constant speed on a substrate with linear heat conduction and no convection, $T_R(x,r) = \exp(-x - \sqrt{x^2+r^2})/\sqrt{x^2+r^2}$, stripped of all constants and where $r = \sqrt{y^2+z^2}$, and the temperature field of the sequence of two such beams moving at a constant distance from each other, $T_{II}(x,r) = T_R(x-1,r) + T_R(x+1,r)$. Then, it is simple to verify that the 2D melt pool trace of the $2/e$-isotherm of $1.6435T_R$ and T_{II} are half-circumferences of approximately equal radii, with the difference due to numerical round-off error. The $2/e$-isotherms of $1.6435T_R$ and T_{II} are depicted in Fig. S.11, showing that the two are strikingly different. However, the differences between the 2D traces are almost indistinguishable. Thus, this is an analytical example of two different temperature fields which, for the same value of T ($2/e$ in this case), have different T-isotherms, but coincident 2D traces.

6 Temperature-dependent material properties

The temperature-dependent properties of 17-4PH SS and Ti-6Al-4V used in the calculations are plotted in Fig. S.12.

References

1. Rosenthal, D. The theory of moving sources of heat and its application of metal treatments. *Transactions ASME* **68**, 849–866 (1946).

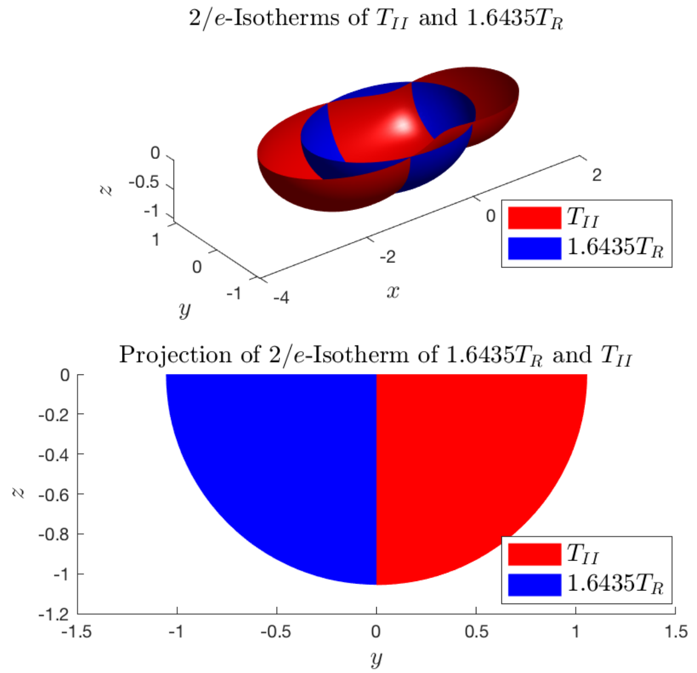

Figure S.11. Top: the 3D shape of the $2/e$-isotherm for $1.6435T_R$ and T_{II}. Bottom: the comparison between the 2D traces of the $2/e$-isotherms for the same temperature fields (only half of each is shown). There is not any visible difference between the two traces.

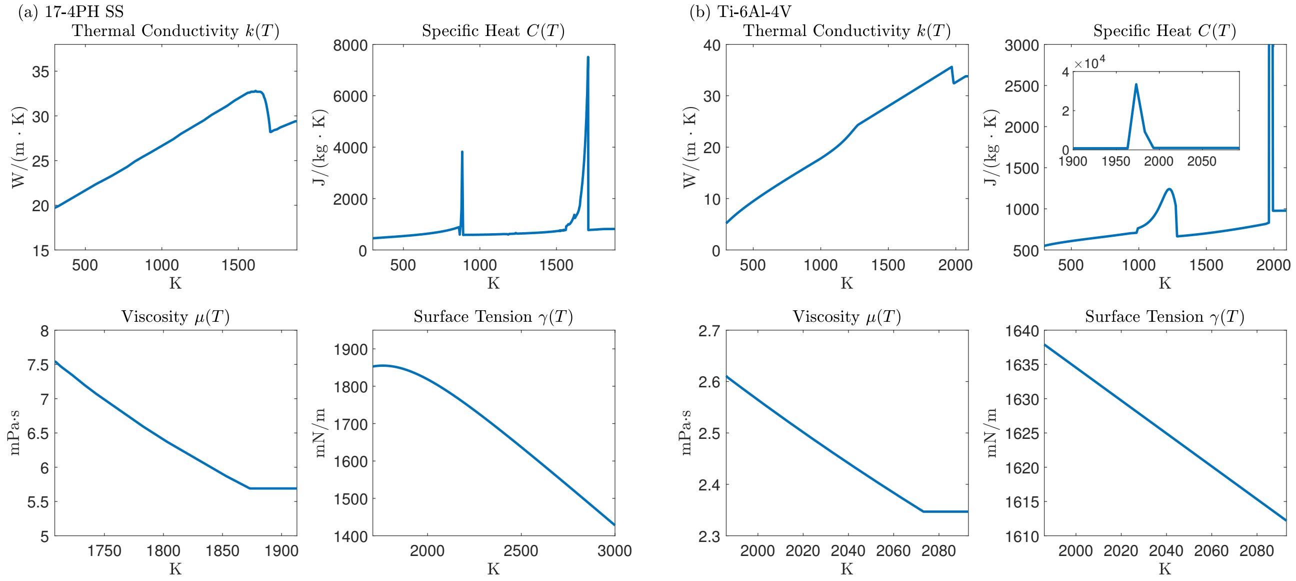

Figure S.12. Temperature dependent material properties. (a) 17-4PH SS (b) Ti-6Al-4V. The adopted values for mass density are 7710 kg/m^3 for 17-4PH SS and 4421 kg/m^3 for Ti-6Al-4V.
